# Investigating the three-dimensional myocardial micro-architecture in the laminar structure using X-ray phase-contrast microtomography

**DOI:** 10.1038/s41598-024-65371-z

**Published:** 2024-06-21

**Authors:** Shunli Wang, Yan Wang, Zhaorui Li, Yifei Zhao, Ying Zhang, François Varray

**Affiliations:** 1 School of Instrumentation Science and Engineering, Harbin Institute of Technology (HIT), Harbin, 150080 China; 2 Department of Medical Engineering, Strategic Support Force Xingcheng Special Duty Sanatorium, Xingcheng, 125100 China; 3 Université de Lyon, CREATIS, CNRS UMR5220, Inserm U1206, INSA-Lyon, Université Lyon 1, 69100 Villeurbanne, France; 4 System Engineering Research Department, Beijing Institute of Aerospace Automatic Controls, Beijing, 100070 China

**Keywords:** Human left ventricular wall, Laminar structure, Cardiomyocyte aggregate arrangement, Myocardial micro-architecture, X-ray phase-contrast microtomography, Anatomy, Cardiology

## Abstract

A comprehensive grasp of the myocardial micro-architecture is essential for understanding diverse heart functions. This study aimed to investigate three-dimensional (3D) cardiomyocyte arrangement in the laminar structure using X-ray phase-contrast microtomography. Using the ID-19 beamline at the European Synchrotron Radiation Facility, we imaged human left ventricular (LV) wall transparietal samples and reconstructed them with an isotropic voxel edge length of 3.5 μm. From the reconstructed volumes, we extracted different regions to analyze the orientation distribution of local cardiomyocyte aggregates, presenting findings in terms of helix and intrusion angles. In regions containing one sheetlet population, we observed cardiomyocyte aggregates running along the local LV wall’s radial direction at the border of sheetlets, branching and merging into a complex network around connecting points of different sheetlets, and bending to accommodate vessel passages. In regions with two sheetlet populations, the helix angle of local cardiomyocyte aggregates experiences a nonmonotonic change, and some cardiomyocyte aggregates run along the local radial direction. X-ray phase-contrast microtomography is a valuable technique for investigating the 3D local myocardial architecture at microscopic level. The arrangement of local cardiomyocyte aggregates in the LV wall proves to be both regional and complex, intricately linked to the local laminar structure.

## Introduction

Cardiomyocytes comprise the main percentage of the heart wall volume, and their arrangement plays a critical role in the cardiac electrophysiology patterns^1^, mechanical functions^2^, and overall heart health^3,4^. Over the years, several conceptual models have been proposed to describe the organization of cardiomyocytes in the left ventricular (LV) wall, including the helical ventricular myocardial band^5^, the nested doughnut (or pretzel)^6^, the three-layered ventricle^7^, and the laminar structure model^8^. The laminar structure model depicts the local myocardial architecture, showing that a network of extracellular collagen fibers organizes cardiomyocytes into sheetlets that are approximately four cells thick^9^. Within the endomysium, collagen fibers are abundant, tightly bundling cardiomyocytes to work together; whereas in the perimysium, sparse collagen fibers allow for relative movements between adjacent sheetlets^10^. A good understanding of the myocardial micro-architecture within the laminar structure enhances our comprehension of sheetlet mechanism and different heart functions.

Laminar architecture has been described using various two-dimensional (2D) imaging techniques such as the optical microscopy of histological sections^11^ and the scanning electron microscopy^9^. While these techniques offer high 2D spatial resolution, they are limited by distortions resulting from the thin sample sectioning. Although these techniques yield important 2D morphological information, they are not fully adapted to reconstruct complex three-dimensional (3D) microstructures that are necessary to characterize the myocardial architecture in detail. Confocal microscopy, with its excellent 3D resolution, is constrained by its inability to capture large image volumes^12^. Magnetic resonance diffusion tensor imaging is widely used to analyze the myocardial fibers architecture, identifying abnormal orientations, and determining local myocardial sheetlet populations^3,13^. While capable of providing in vivo 3D images, its spatial resolution is insufficient for accurately visualizing the local myocardial microstructure.

To address these constraints, a novel high-resolution 3D X-ray imaging technique, known as synchrotron radiation phase-contrast microtomography (SR-PCT), has been introduced for the study of myocardial micro-architecture^8,14–17^. In comparison to conventional X-ray attenuation-based imaging, the X-ray phase-contrast imaging exhibits significantly higher sensitivity in biological media^18,19^. SR-PCT can image tissue structures of several cubic centimeters in size at a resolution of a few microns, providing data with histology-like contrast^20,21^. Most tissue components, such as cardiomyocytes, coronary vessels, elastin, sheetlets, and the epicardium, are visible in the reconstructed volumes^8,16,22^. Nevertheless, existing quantitative studies have predominantly focused on the large-scale distribution of the orientation of cardiomyocyte aggregates. There remains a gap in research concerning the local 3D myocardial architecture.

In contrast to our prior investigations into the laminar structure in samples^8,23,24^, this study specifically examines different volumes from various laminar structure regions across different samples. Our focus here is on delineating the 3D myocardial micro-architecture within these volumes at the resolution of individual cells. In the Material and Methods section, we present the data acquisition step and the strategy for measuring the orientation of local cardiomyocyte aggregates. In the Results section, we manually select volumes to depict the local myocardial micro-architecture in the laminar structure and present the 3D distribution of the orientation of cardiomyocyte aggregates in terms of the helix angle (HA) and intrusion angle (IA). Finally, the Discussion and Conclusion sections end the paper.

## Material and methods

This study was conducted in accordance with the principles of the Declaration of Helsinki, and the informed consent was obtained from the subjects’ legal guardians. All methods were carried out in accordance with relevant guidelines and regulations, and the experimental protocols were approved by the medical ethics committee of Harbin Institute of Technology.

### Sample collection and preservation

**Figure 1:**
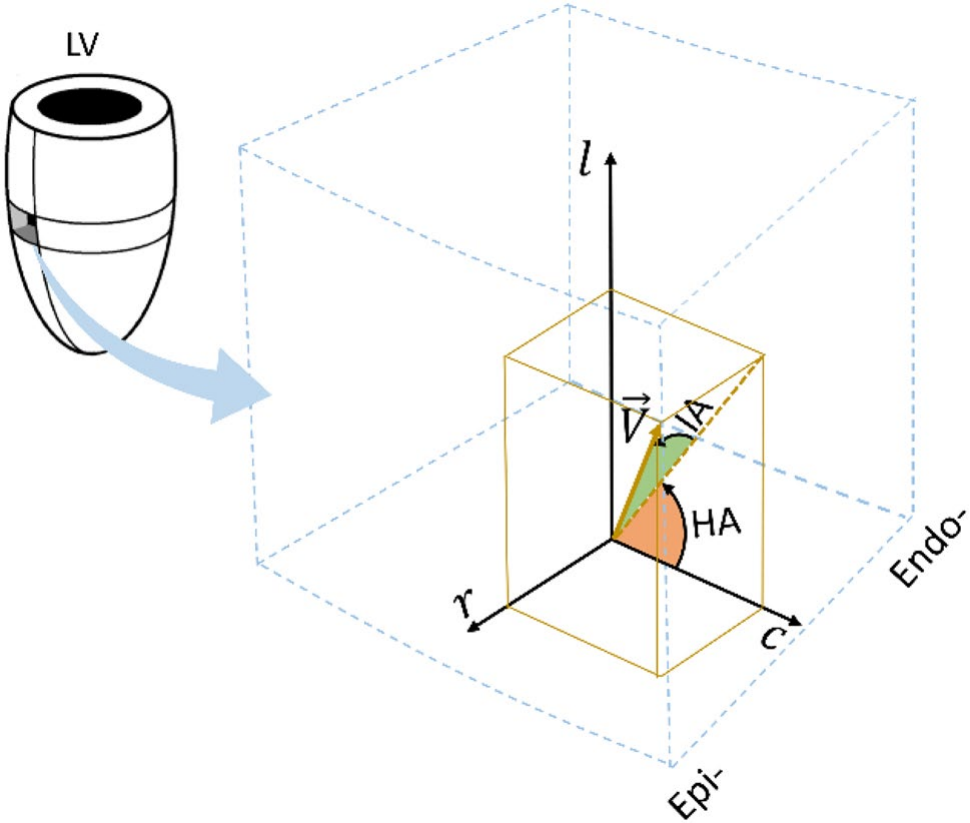
Definition of the helix angle (HA) and intrusion angle (IA) in the local $(r,c,l)$ coordinates. In the $(r,c,l)$ coordinates, axes $r$, $c$, $l$ correspond to the local left ventricular wall’s radial, circumferential and longitudinal directions, respectively. $\overrightarrow{V}$ denotes the orientation of local structure.

**Figure 2:**
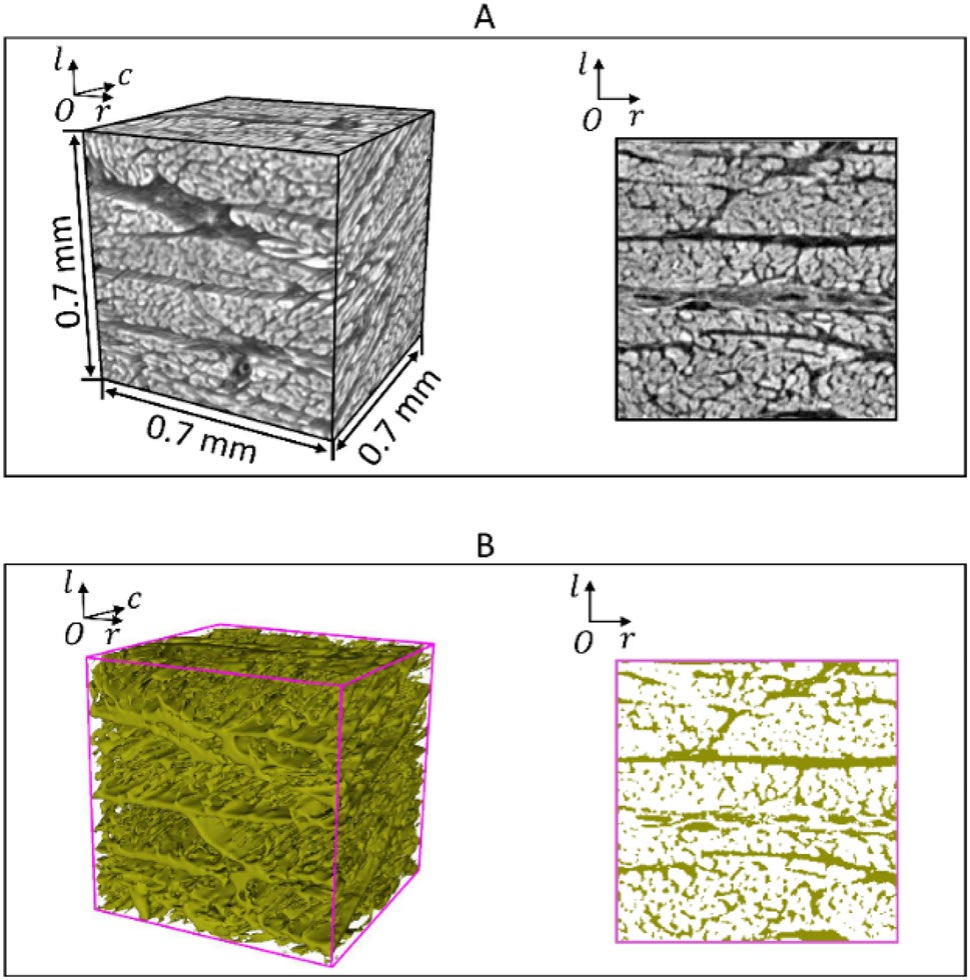
Extraction of the extracellular matrix from the myocardial tissue. (**A**) Reconstructed data after suppressing the low-frequency variation of the background density. (**B**) Extracellular matrix segmented from the volume.

**Figure 3:**
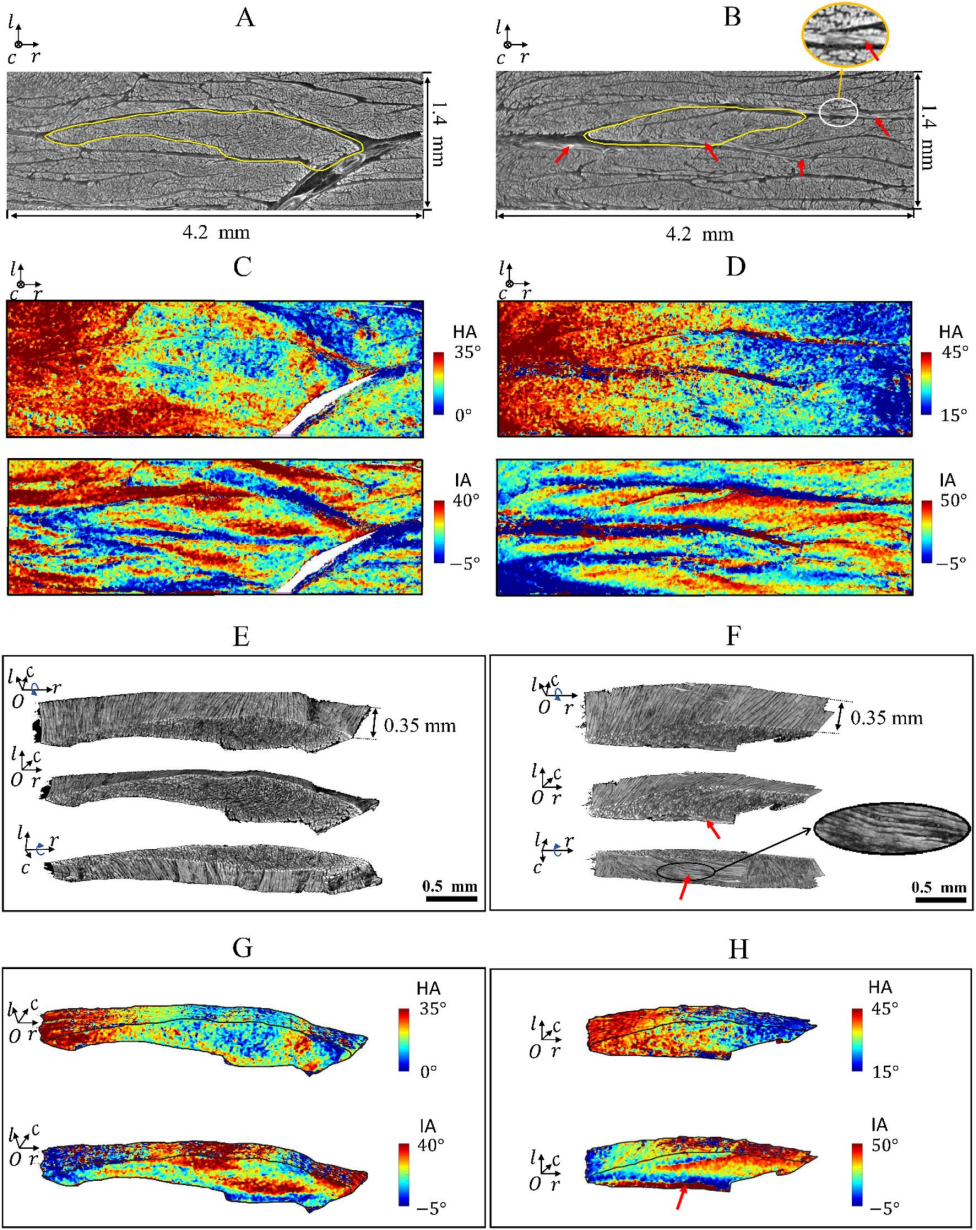
Arrangement of cardiomyocyte aggregates in the volumes containing a single population of sheetlets. (**A**, **B**) Raw reconstructed data in the volumes. Red arrows point to the cardiomyocytes running along the local $r$-direction. A sheetlet is manually selected from each volume, highlighted by a yellow outline. (**C**, **D**) Distribution of the cardiomyocyte HA and IA in the volumes. (**E**, **F**) Cardiomyocyte aggregates in the selected sheetlets. (**G**, **H**) Distribution of the cardiomyocyte HA and IA in the selected sheetlets.

**Figure 4:**
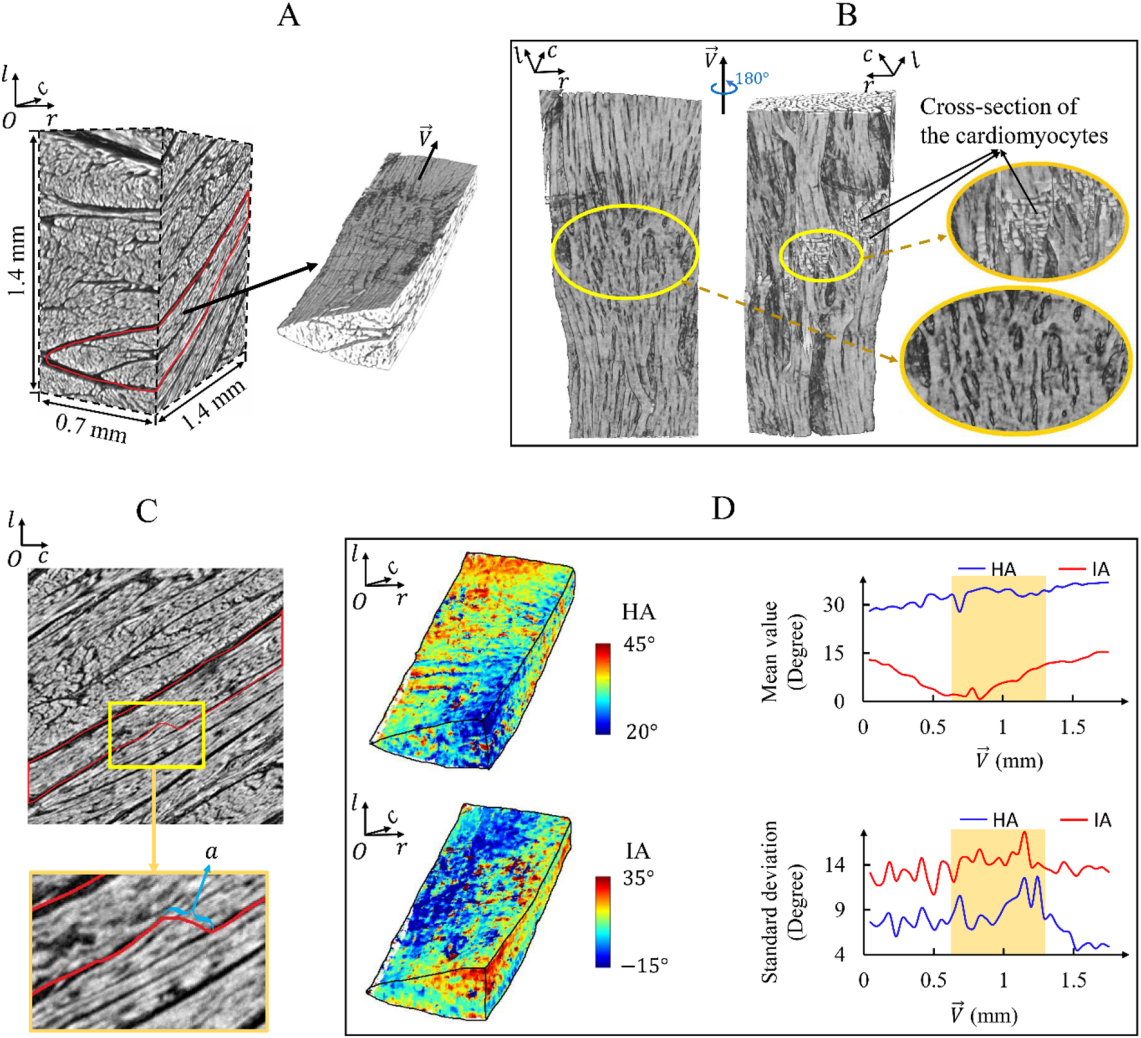
Arrangement of cardiomyocyte aggregates around the connecting region of different sheetlets. (**A**) Raw reconstructed data with $\overrightarrow{V}$ denoting the mean orientation of the cardiomyocyte aggregates in a selected sheetlet. (**B**) Cardiomyocyte aggregates in the selected sheetlet. The connecting region of different sheetlets is marked by using a yellow circle. (**C**) Raw reconstructed data in the volume’s mid-$cOl$ section. The red outline corresponds to the border of the selected sheetlet. At location ‘a’, cardiomyocyte aggregates are cut off, which causes the cross-section of cardiomyocytes in (**B**). (**D**) Distribution of local cardiomyocyte HA and IA in the selected sheetlet, and profile of their mean values and standard deviations along the $\overrightarrow{V}$ direction.

**Figure 5:**
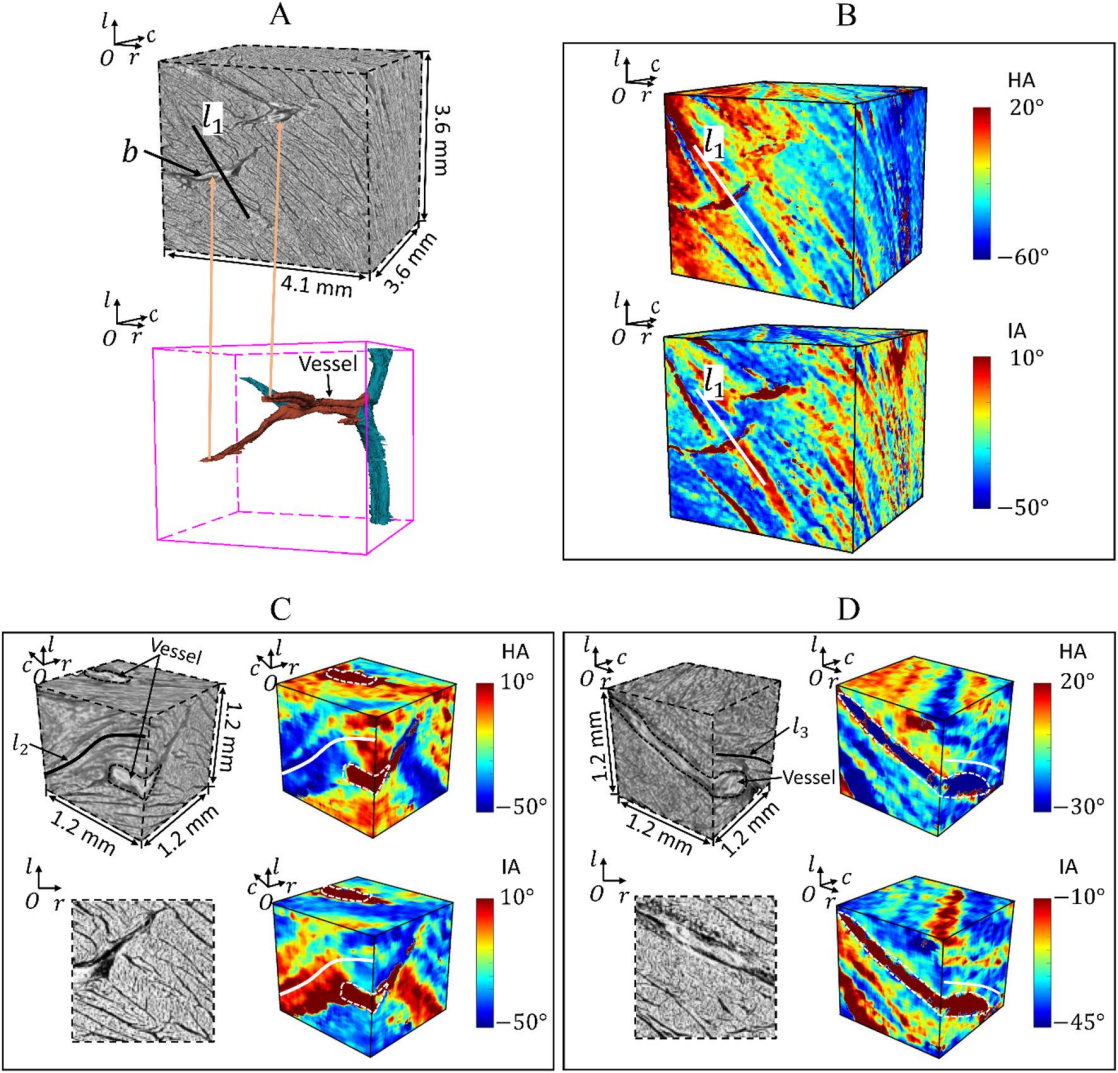
Arrangement of cardiomyocyte aggregates around a big vessel. (**A**) Raw reconstructed data (upper image) and the main vessels (lower image) in the volume. The red vessels intersect with nearby sheetlets, while the blue vessels lie parallel to their nearby sheetlets. (**B**) Distribution of the orientation of local cardiomyocyte aggregates in the volume, showcasing HA and IA. Arrangement of cardiomyocyte aggregates in two sub-volumes: (**C**) the vessel intersects with nearby sheetlets and (**D**) the vessel is almost parallel to nearby sheetlets.

**Figure 6:**
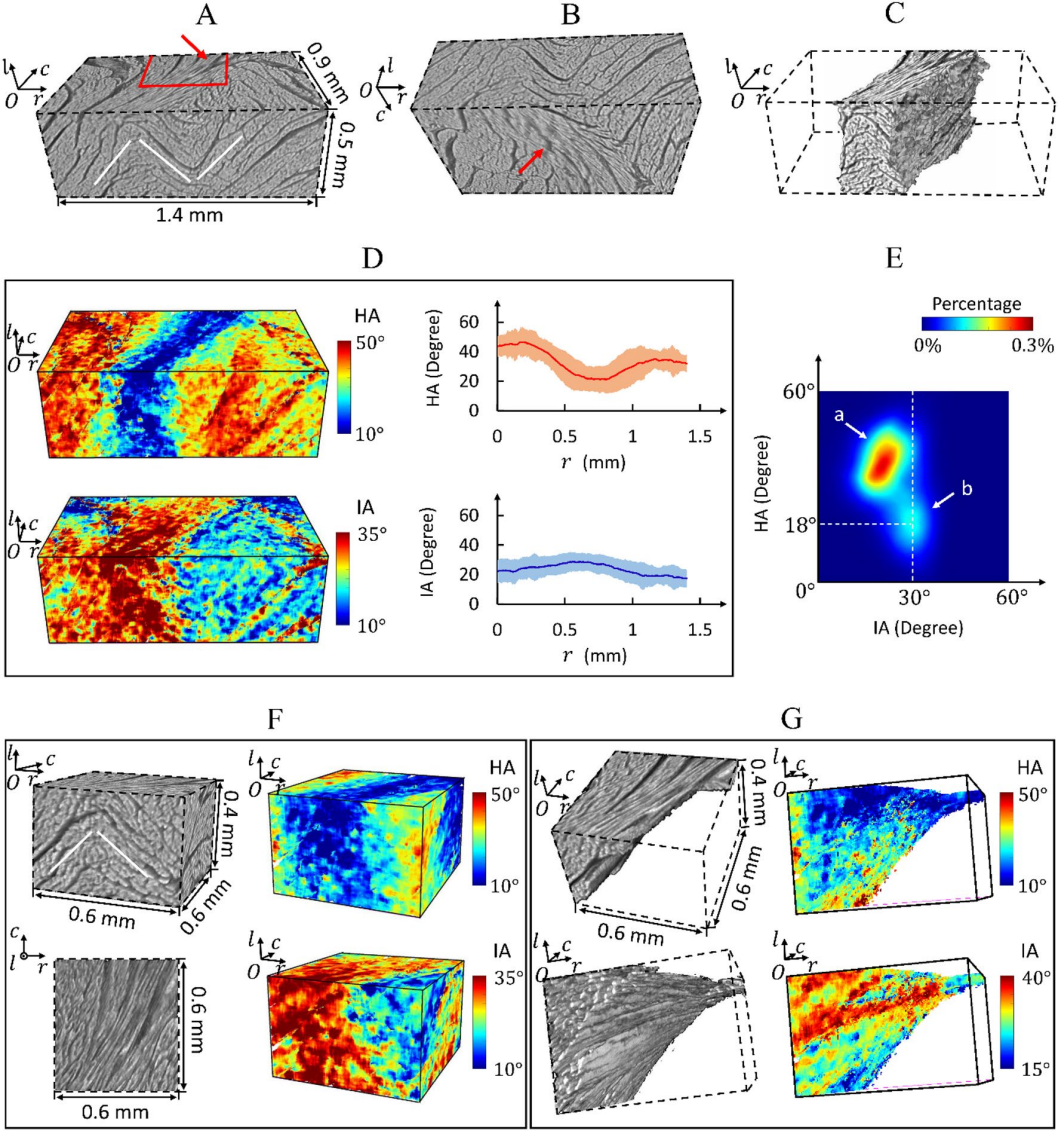
Arrangement of cardiomyocyte aggregates in a volume containing two sheetlet populations, where the two populations alternate along the local $r$-direction. (**A**, **B**) Raw reconstructed data in the volume. The white lines follow the arrangement of local sheetlets. The red lines correspond to the border of the sub-volume in (**F**). The red arrow points to the cardiomyocyte aggregates whose helix angle is small. (**C**) Cardiomyocyte aggregates with helix angle smaller than $18^\circ$. (**D**) Local cardiomyocyte HA and IA in the volume, and the profiles of their values (including mean value, standard deviation) along the local $r$-direction. (**E**) Joint distribution of cardiomyocyte HA and IA in the volume. The orientation of the local cardiomyocyte aggregates belongs to two separate classes (named “a” and “b”). (**F**, **G**) Cardiomyocyte aggregates and their local HA and IA in the sub-volume and sub-sub-volume. The sub-sub-volume is manually selected from the sub-volume in (**F**).

**Figure 7:**
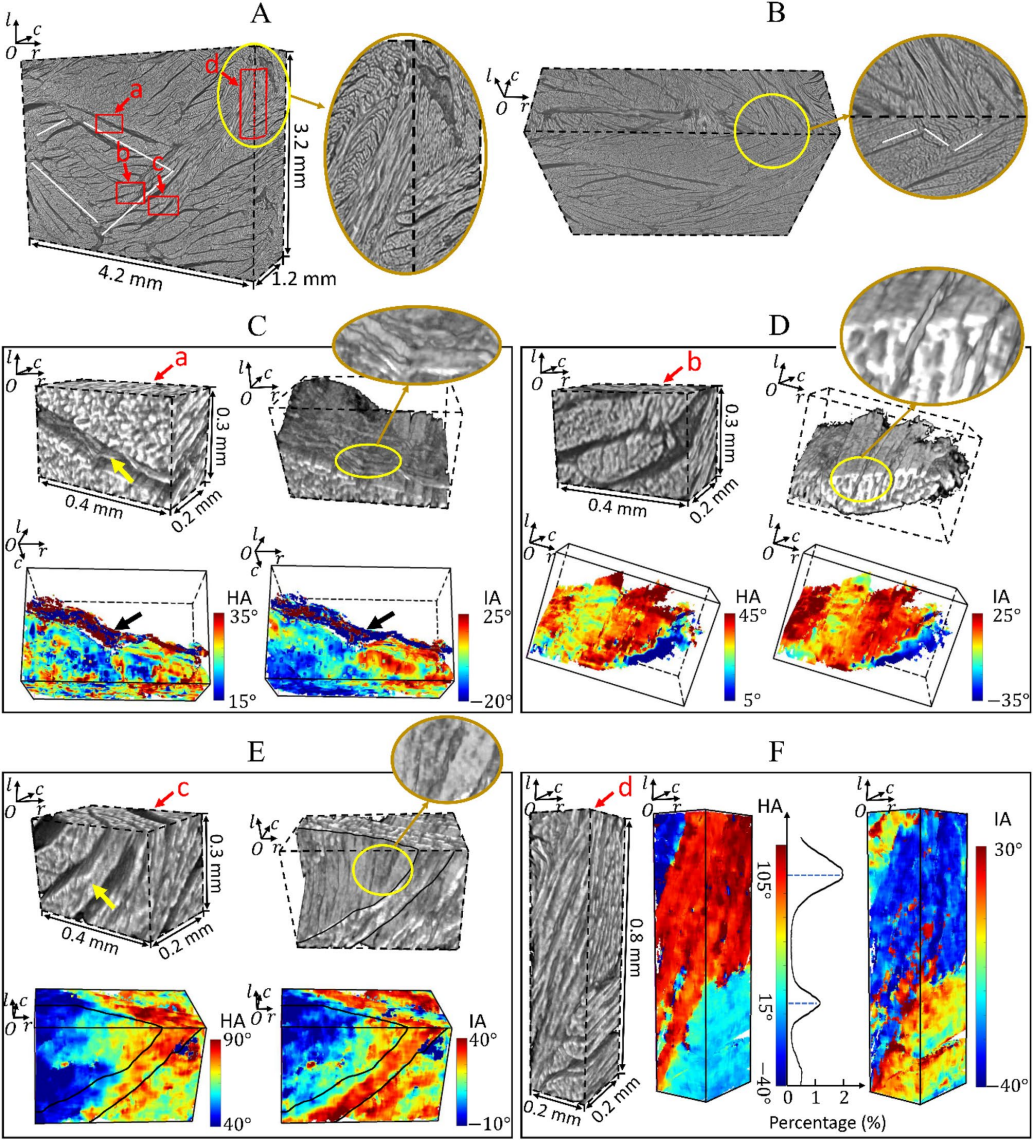
Arrangement of cardiomyocyte aggregates in a volume containing two sheetlet populations, where the two sheetlet populations are organized into a parallelogram-like pattern. (**A**, **B**) Raw reconstructed data in the volume. White lines depict the arrangement of the two sheetlet populations. Four sub-volumes are outlined with red borders. (**C**, **D**, **E**, **F**) Arrangement of the cardiomyocyte aggregates in the four sub-volumes. From these sub-volumes, local regions are further manually extracted to illustrate the local cardiomyocyte arrangement.

The Medico-Legal Institute of Lyon supplied eight human heart samples (No. DC-2012–1588). These human hearts were extracted from two males, aged 32 and 35 years, without any history of heart disease or addiction, who died accidentally. Within 24 h of the patients’ death, their hearts were extracted and immersed in a 10% formalin solution for preservation before the postmortem examination. This preservation step was carried out to maintain optimal tissue architecture (Figs. 1–7).

**Figure 8:**
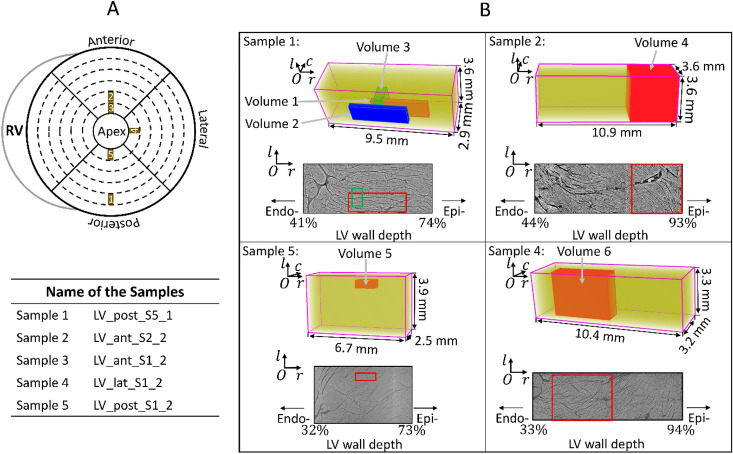
Location of the volumes in LV wall. (**A**) Locations and names of five transparietal samples from LV wall. (**B**) Volumes in the samples’ laminar structure regions. The laminar structure regions (in yellow) coincide with that in literature ^8,24^. The red (or blue) outlines in the two-dimensional $rOl$-sections correspond to the borders of the volumes.

Transparietal samples were manually extracted from the short-axis slices of the left ventricles (Fig. 8 in Appendix), which crossed the local wall from the endocardium to the epicardium. The lengths of the samples varied, which was related to their location in the LV wall and heart size. The sample sizes along both the local longitudinal and circumferential directions were approximately 5 mm. Further details can be found in literature^8,22,24^.

### Image acquisition

To enhance tissue contrast in X-ray projections and make the sheetlets (and cleavage planes) visible in the reconstructed volumes, we subjected the samples to a rehydration and dehydration process before imaging. In the rehydration step, the samples were rinsed with deionized water and plunged into demineralized water. In the ethanol gradient dehydration step, the samples were sequentially immersed in several ethanol solutions, with each bath lasting approximately 30 min. The ethanol concentration in the baths increased from 10 to 70% in 10% increments.

At the ID-19 beamline of the European Synchrotron Radiation Facility (ESRF) in Grenoble France, we fixed each sample-containing tube upright on the translation-rotation stage for tomographic imaging. In each tube, beside the sample, there do not exist other things such as liquid solution. The X-ray beam energy used was an unfiltered first-harmonic undulator radiation at 19 keV, with an energy bandwidth of approximately 1%. The signal detector comprised standard microscope optics and a high-speed low-noise CCD-based camera featuring a chip with dimensions of 2048 × 2048 pixels, each measuring 14 × 14 μm^2^. This detector was situated 750 mm downstream of the sample for phase-contrast, with a lens magnification about 4^25^. During each scan, the sample was projected from 2499 uniformly distributed angles, with a counting time of 0.4 s for each projection and about 20 min for whole scan. Due to the detector’s limited field-of-view (3.5 mm or 7 mm in height), successive tomographic scans were conducted to cover each sample (16 mm to 28 mm in length). The adjacent scans contained an overlap of 100 voxels (about 0.35 mm) to prevent intensity discontinues between the reconstructed sub-volumes.

We applied Paganin’s algorithm for a phase at each projection angle^26^. In this algorithm, the object was assumed to be homogeneous, and the $\delta /\beta$ parameter was set to 900. Subsequently, we used the filtered back-projection method to reconstruct the projections from each scan into a volume of 2048 × 2048 × 2048 voxels in size, with an isotropic voxel edge length equaling 3.5 μm. Following the removal of the overlapping slices, the transparietal samples comprised substantial amounts of data, with the size of each SR-PCT reconstructed volume ranging from 60 to 80 GB.

### Measurement of the orientation of local cardiomyocyte aggregates

The reconstructed sample volumes were analyzed in local $(r,c,l)$ coordinates, where the axes $r$, $c$ and $l$ correspond to the local LV wall’s radial, circumferential and longitudinal directions, respectively. To investigate the regional arrangement of the cardiomyocyte aggregates within the laminar structure, we extracted small volumes from the samples and assessed the orientation distribution of the local cardiomyocyte aggregates inside.

For each voxel in the selected volumes, we measured the local structure orientation using the gradient structure-tensor method proposed by literature^16^, with the following steps:

(1) Use a central difference algorithm to compute the local gradient magnitudes along the local LV wall’s radial, circumferential and longitudinal directions (denoted as ${I}_{r}$, ${I}_{c}$ and ${I}_{l}$), respectively.

(2) Define the local structure tensor $ST$ in the voxel as:

1 $$ST={\left[\begin{array}{ccc}{I}_{r}& {I}_{c}& {I}_{l}\end{array}\right]}^{T}\cdot \left[\begin{array}{ccc}{I}_{r}& {I}_{c}& {I}_{l}\end{array}\right]=\left[\begin{array}{ccc}{I}_{r,r}& {I}_{r,c}& {I}_{r,l}\\ {I}_{c,r}& {I}_{c,c}& {I}_{c,l}\\ {I}_{l,r}& {I}_{l,c}& {I}_{l.l}\end{array}\right]$$

where $T$ is the matrix transpose operator, and ${I}_{i,j}={I}_{i}\times {I}_{j}$ with $(i,j)$ in $(r,c,l)$.

(3) Flatten each component in the structure tensor using an average filter:

2 $$\overline{ST }=\left[\begin{array}{ccc}\overline{{I }_{r,r}}& \overline{{I }_{r,c}}& \overline{{I }_{r,l}}\\ \overline{{I }_{c,r}}& \overline{{I }_{c,c}}& \overline{{I }_{c,l}}\\ \overline{{I }_{l,r}}& \overline{{I }_{l,c}}& \overline{{I }_{l,l}}\end{array}\right]$$

where $\overline{{I }_{r,r}}$ is the mean value of ${I}_{r,r}$ in a sub-volume with a size of 5 × 5 × 5 voxels and centered on the voxel under measurement.

(4) Decompose the flattened structure tensor $\overline{ST }$ into a set of eigenvectors and eigenvalues, and regard the tertiary eigenvector (i.e. the eigenvector corresponding to the smallest eigenvalue) as the orientation $\overrightarrow{V}=({v}_{r},{v}_{c},{v}_{l})$ of the local structure in the voxel.

(5) Calculate the values of the local structure’s helix angle (HA) and intrusion angle (IA)^23,27^ in the voxel based on the definitions in Fig. 1:

3 $$\left\{\begin{array}{c}HA=arctan\left(\frac{{v}_{l}}{{v}_{c}}\right)\\ IA=arctan\left(\frac{{v}_{r}}{\sqrt{{{v}_{c}}^{2}+{{v}_{l}}^{2}}}\right)\end{array}\right.$$

Additional details, including the “Location of the volumes in the left ventricular wall” and the “Illustration of the gradient structure-tensor method in measuring the orientation of local myocardial structures”, can be found in the Appendix section.

### Extraction of the extracellular matrix from myocardial tissue samples

Within the LV wall, the cardiomyocytes are embedded in the extracellular matrix (ECM) to organize the myocardium (Fig. 2A), where the ECM includes the endomysium and the epimysium (Fig. 2B). To investigate the distribution of the orientation of local cardiomyocyte aggregates, the ECM was extracted with the following two steps and the structure tensor inside was removed:

- (1) Suppress the gradual variations of background density in the reconstructed volumes using a cubic-centered Gaussian filter $G(0,\sigma =44)$, where the parameter $\upsigma$ is large enough to preserve both the cardiomyocyte aggregates and ECM^8^;
- (2) Separate the cardiomyocyte aggregates from the ECM in the reconstructed volumes using the Otsu’s method (i.e. a thresholding technique).

## Results

In the samples, most cardiomyocyte aggregates are organized into laminar structures, where the local sheetlets may belong to either one population or two different populations^8,24^. From the laminar structure regions, we manually selected several volumes as the regions of interest to present the regional 3D myocardial micro-architecture at a cell-level resolution.

### Volumes containing one population of sheetlets

Figure 3 illustrates the arrangement of local cardiomyocyte aggregates in volumes containing a single population of sheetlets. Both the volumes are 400 × 1200 × 100 voxels in size, corresponding to 1.4 × 4.2 × 0.35 mm^3^ (Fig. 3A,B). In the volumes, the cardiomyocyte HA decreases along the $r$-direction, whereas the IA oscillates along the $l$-direction (Fig. 3C,D).

From each volume, one sheetlet was manually extracted to depict the local cardiomyocyte arrangement (Fig. 3E,F). It was observed that in most sheetlets, the local cardiomyocytes are approximately parallel (Fig. 3A,E). However, at the border of some sheetlets, the cardiomyocytes bend to be almost perpendicular to those inside the sheetlets (indicated by a red arrow in Fig. 3B,F,H). Figure 3G,H presents the distribution of the orientation of local cardiomyocyte aggregates in the selected sheetlets. In both cases, the HA value decreases gradually along the $r$-direction, and the IA value is regionally variable, demonstrating an irregular distribution. For the cardiomyocyte aggregates running along the local $r$-direction, their absolute IA value is larger than that in the surrounding area (Fig. 3D,H).

In the volume presented in Fig. 4A, only one sheetlet population is observed. A sheetlet was selected to showcase the arrangement of the cardiomyocyte aggregates around the connecting part of two adjacent sheetlets (Fig. 4B). Figure 4C shows the raw reconstructed data in the volume’s mid-$cOl$ plane. The red outline corresponds to the border of the selected sheetlet. At location ‘$a$’, the cardiomyocyte aggregates are cut off, resulting in the cross-section of cardiomyocytes shown in Fig. 4B.

Around the connecting region of different sheetlets, we observed that the cardiomyocyte aggregates branch and merge into a complex network (Fig. 4B). This pattern differs from the relatively parallel alignment of cardiomyocyte aggregates in most other regions. Consistent with the tissue micro-architecture, around the connecting region of different sheetlets, the standard deviations of the cardiomyocyte HA and IA are larger than those in the other parts of the sheetlet (Fig. 4D).

Beside the sheetlets, there may exist other large components, such as the coronary vessel (Fig. 5A), in the laminar structure region. Locally, the relative position between the vessel and the sheetlets presents two situations: a vessel intersecting with nearby sheetlets (rendered in red) or a vessel running parallel to nearby sheetlets (rendered in blue). The myocardial architecture around the vessels is complex. In Fig. 5A,B, avessel’s cross-section on the first $rOl$-plane is marked as ‘$b$’; passing ‘$b$’, a line ‘${l}_{1}$’ is drawn, which is parallel to the local sheetlets. Along the line ‘${l}_{1}$’, both cardiomyocyte HA and IA experience a sudden change.

To observe the local cardiomyocyte aggregates around the vessel, we zoomed into two sub-volumes. In Fig. 5C, the vessel intersects with local sheetlets; while in Fig. 5D, the vessel is almost parallel to local sheetlets. Lines ‘${l}_{2}$’ and ‘${l}_{3}$’ are drawn to show the local helical orientation of cardiomyocyte aggregates around the vessels. In both these cases, we note that around the vessel, the cardiomyocyte aggregates consistently bend to provide space for the vessel to pass.

### Volumes containing two populations of sheetlets

Figure 6 depicts the arrangement of cardiomyocyte aggregates in a volume containing two populations of sheetlets, where the two populations alternate along the LV wall’s local $r$-direction (Fig. 6A,B). Around the two populations’ intersection part, the cardiomyocyte HA value is smaller than the surroundings, and the cardiomyocyte IA value is larger (Fig. 6C,D). Figure 6E presents the joint distribution of cardiomyocyte HA and IA in the volume, revealing two distinct classes of cardiomyocyte orientation. Figure 6C depicts the orientation of local cardiomyocyte aggregates around the two sheetlet populations’ intersection part belonging to the class ‘$b$’ in Fig. 6E.

To clearly observe the arrangement of local cardiomyocyte aggregates around the intersection part of two sheetlet populations, a sub-volume was extracted (Fig. 6F), and a small part was further selected from the sub-volume (Fig. 6G). In Fig. 6F,G, we note that the cardiomyocyte aggregates belonging to two sheetlet populations are not separate. They connect in the way of sheetlets’ branching and merging.

In certain regions, the two sheetlet populations exhibit a more complex arrangement, forming a parallelogram-like pattern, as depicted in Fig. 7A,B. Around the regions with a parallelogram-like pattern, there may also exist an alternating pattern of two sheetlet populations shown in Fig. 6A.

From the volume depicted in Fig. 7A, four sub-volumes were extracted and magnified to show the local cardiomyocyte arrangement. In the sub-volume in Fig. 7C, we note that between the adjacent sheetlets, there are the cardiomyocytes running along the local $r$-direction, approximately perpendicular to the cardiomyocytes in the sheetlets. Different from the cardiomyocytes pointed by a red arrow in Fig. 3F, the cardiomyocytes pointed by a yellow arrow here are sparser and more curved. Figure 7D illustrates the myocardial micro-architecture around the intersection part of the sheetlet populations, where the sheetlets have more cracks. Figure 7E shows that in some sheetlets, the orientation of local cardiomyocyte aggregates undergoes a significant change. In the sheetlet indicated using a yellow arrow, the orientation of local cardiomyocyte aggregates changes from the local $cOl$-plane to the local $rOl$-plane, with an increase in both HA and IA. Figure 7F depicts the local cardiomyocyte aggregates almost perpendicularly.

## Discussion

To investigate the cardiomyocyte arrangement in the human heart, we utilized SR-PCT to image a set of human LV free wall transparietal samples at the ESRF in Grenoble France. Previous studies have detailed the transmural distribution of the orientation of local cardiomyocyte aggregates in the samples^8,22–24^. In this study, we manually selected volumes from the laminar structure regions of different samples (Fig. 8 in the Appendix), and delineated the 3D myocardial micro-architecture inside these volumes at cell-level resolution Figs. (3–7).

### Result analysis

During the samples’ preservation and imaging-preparation steps, the use of formalin fixation and ethanol gradient dehydration may lead to sample volume shrinkages, causing a subtle gradual change in tissue architecture^28,29^. Most tissue components are visible^8,24^ in the reconstructed SR-PCT volumes with an isotropic voxel edge length of 3.5 μm. Mirea et al*.* validated the arrangement of local cardiomyocyte aggregates by comparing orthogonal sections with optical images of several histological sections, affirming that the myocardial architecture in the volumes was in good condition^20^. Importantly, the volumes under study were extracted from the interior of the samples to avoid the edge distortion, ensuring the reliability of the myocardial architecture observed in these volumes.

To determine the orientation of local cardiomyocyte aggregates, we opted for the gradient structure-tensor method over the Fourier-based method, taking into account their working window sizes. To the data used in this paper, the smallest size of working window in Fourier-based method is 32 × 32 × 32 voxels (i.e. 112 × 112 × 112 μm^3^)^22^, which determines that the Fourier-based method can only be used to measure the mean orientation of several hundred cardiomyocytes. The working window in the gradient structure-tensor method can be extremely small, for example 3 × 3 × 3 voxels. However, to reduce the influence of noise, we used an average filter (5 × 5 × 5 voxels in size) to flatten the components in the measured structure tensor. This approach ensures that the results of the gradient structure-tensor method more accurately reflect the local orientation of cardiomyocyte aggregates. The IA was selected to describe the radial orientation of local cardiomyocyte aggregates, avoiding the projection effect^23,27^.

In the regions containing just one population of sheetlets, we note that the orientation of cardiomyocyte aggregates within single sheetlet is heterogeneous (Fig. 3). In addition to the gradual change of HA along the $r$-direction, the cardiomyocyte IA is far from constant (Fig. 3G,H). In literature^23,24^, it was pointed out that the IA may experience an oscillation along the normal direction of local sheetlets. However, this hypothesis remains controversial. Notably, at the border of some sheetlets, we observed cardiomyocyte aggregates running along the $r$-direction, perpendicular to the nearby cardiomyocytes inside the sheetlet (Fig. 3B,F).

In the regions containing two populations, sheetlets may organize into a serration-like pattern (Fig. 6) or a parallelogram-like pattern (Fig. 7). Both patterns are deformable and can contribute to the LV wall’s systolic thickening through the sheetlet rearrangement. However, the myocardial architecture in these regions is complex, and the relationship between these structures to the local LV mechanical functions remains unclear.

### Limitation

Current SR-PCT is far from wide clinical application, due to its large size, limited access (i.e. high cost, few time slots), and strict imaging conditions (i.e. small field of view, long acquisition time, and high X-ray energy). In the field of heart studies, it is still mainly used to image ex vivo tissue samples or the hearts of small animals. Owing to the heart’s unclear contraction state, it is difficult to predict the evolution of the local myocardial micro-architecture during a heart circle.

### Clinical perspectives

With the rapid development of laboratory phase-contrast microtomography, we anticipate the imminent maturation of X-ray phase-contrast tomography as a robust medical imaging technology. The findings from this study endorse the application of clinical X-ray phase-contrast tomography for the diagnosis of myocardial diseases.

Furthermore, delving into myocardial micro-architecture enhances our comprehension of the cardiac mechanism and facilitates the construction of an accurate heart structural model. This knowledge, in turn, can catalyze advancements in other cardiac imaging techniques, such as the diffusion tensor imaging and the echocardiography.

## Conclusion

Using X-ray phase-contrast microtomography, we explored the 3D local myocardial architecture within the human LV laminar structure at microscopic level. As far as we known, this is the first time when the regional 3D arrangement of cardiomyocyte aggregates in the laminar structure is clearly observed. Our results present the advantage of SR-PCT in the histomorphological study of human left ventricle, paving the way for future endeavors to deepen our insight into myocardial functions.

## Figures and Tables

**Figure 9:**
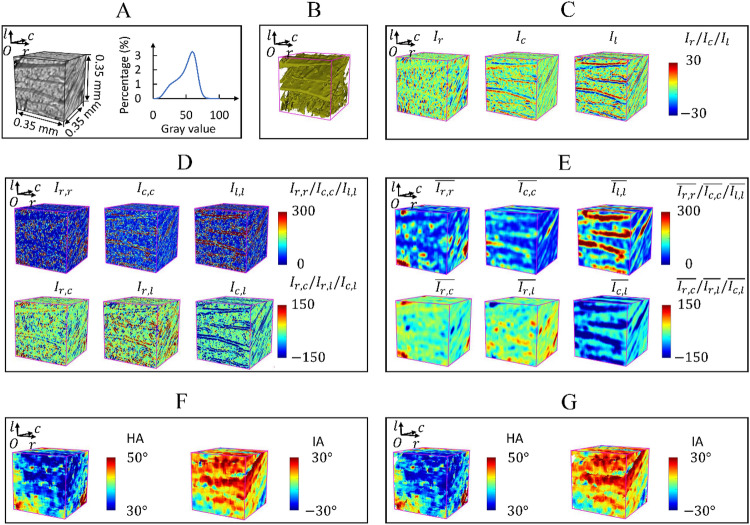
Processing strategy of the gradient structure-tensor method in measuring the orientation of local myocardial aggregates. (**A**) Raw reconstructed data in a volume of 0.35 × 0.35 × 0.35 mm^3^ in size. (**B**) The extracted extracellular matrix (ECM) in the volume. (**C**) Distribution of the measured ${I}_{r}$, ${I}_{c}$ and ${I}_{l}$ in the volume, where ${I}_{r}$, ${I}_{c}$ and ${I}_{l}$ denote the local gradient magnitudes along the local LV wall’s radial, circumferential and longitudinal directions, respectively. (D, E) Distribution of the ${I}_{r,r}$, ${I}_{c,c}$, ${I}_{l,l}$, ${I}_{r,c}$, ${I}_{r,l}$, ${I}_{c,l}$, and $\overline{{I }_{r,r}}$, $\overline{{I }_{c,c}}$, $\overline{{I }_{l,l}}$, $\overline{{I }_{r,c}}$, $\overline{{I }_{r,l}}$, $\overline{{I }_{c,l}}$ in the volumes. The definition of these parameters can be seen in Eq. (1) and Eq. (2). (**F**, **G**) Distribution of HA and IA in the volume. In (**G**), the value of HA and IA in ECM is removed.

## Data Availability

The data underlying this article will be shared upon reasonable request from the corresponding authors.

## Appendix

### Location of the volumes in the left ventricular wall

As outlined in the Material and Methods section, each isolated human heart was divided into six short-axis slices with similar thicknesses of about 5 mm. The transparietal samples were then extracted from these slices. Figure 8A shows the locations of the five samples from the LV free wall, whose numbers and names coincide with those in our previously published papers^8,22–24^. In literature^8,24^, a laminar structure region was extracted from each sample to investigate the arrangement of local cardiomyocyte aggregates (Fig. 8B). This study further selected six volumes from the laminar structure regions to delineate the reconstructed data Volumes 1 and 2 described in Figs. 3A,B, while the Volumes 3 to 6 are presented in Figs. 4–7. Additionally, Fig. 8B provides a 2D $rOl$-section from each laminar structure.

### Illustration of the gradient structure-tensor method in measuring the orientation of local myocardial structures

In the SR-PCT reconstructed volume of the myocardial tissue, the gray values of different components are different (see Figs. 2–9), which indicates that the orientation of the local tissue structure can be measured using the Fourier-based method^8,22–24^ or the gradient structure-tensor method^16^. Figure 9 presents the processing strategy of the gradient structure-tensor method with a volume of 0.35 × 0.35 × 0.35 mm^3^ in size, whose steps have been developed in the section of Material and Methods.

As shown in Fig. 9A, we manually adjusted the data’s gray value range to [0, 100]. Figure 9B shows the extracellular matrix (ECM) in the volume extracted using the strategy presented in Fig. 2. Using the measured local gradient magnitude (Fig. 9C,D,E), the orientation of the local myocardial structure was calculated, and both the helix angle (HA) and intrusion angle (IA) were extracted. In Fig. 9G, we removed the HA and IA value from the ECM.
